# 鳞状上皮细胞癌抗原水平新检测方法在宫颈癌、肺癌和头颈部癌鉴别诊断中的性能及临床表现

**DOI:** 10.3779/j.issn.1009-3419.2018.07.12

**Published:** 2018-07-20

**Authors:** Stefan HOLDENRIEDER, Rafael MOLINA, Ling QIU, Xiuyi ZHI, Sandra RUTZ, Christine ENGEL, Pia KSPER-SAUER, Farshid DAYYANI, Catharina Mkorse

**Affiliations:** 1 波恩大学医院，波恩，德国 University Hospital Bonn, Bonn, Germany; 2 巴塞罗那医院和巴塞罗那大学，巴塞罗那，西班牙 Hospital Clinic de Barcelona and University of Barcelona, Barcelona, Spain; 3 北京协和医院，北京，中国 Peking Union Medical College Hospital, Beijing, China; 4 首都医科大学宣武医院，北京，中国 Xuanwu Hospital, Capital Medical University, Beijing Shi, China; 5 罗氏诊断公司，Penzberg，德国 Roche Diagnostics GmbH, Penzberg, Germany; 6 罗氏诊断公司，Mannheim，德国 Roche Diagnostics GmbH, Mannheim, Germany; 7 罗氏诊断国际有限公司，Rotkreuz，瑞士 Roche Diagnostics International Ltd, Rotkreuz, Switzerland; 8 加利福尼亚大学，Irvine，CA，美国 University of California, Irvine, Irvine, CA, USA; 9 荷兰癌症研究院，阿姆斯特丹，荷兰 The Netherlands Cancer Institute, Amsterdam, The Netherlands

**Keywords:** 鳞状上皮细胞癌, 免疫检测, 颈部, 肺部, 头部, 颈部

## Abstract

鳞状上皮细胞癌抗原水平在鳞状上皮细胞癌中通常升高。这项多中心研究评估了一种新的Elecsys^®^鳞状上皮细胞癌检测的检测性能，该检测方法是以等摩尔方式测量血清鳞状上皮细胞癌抗原1和2的水平，并研究了鳞状上皮细胞癌抗原用于宫颈癌、肺癌和头颈部鳞状上皮细胞癌鉴别诊断的潜能。在欧洲三个研究中心进行了精确度和方法学比较实验。健康人群的参考区间使用来自欧洲和中国人群的样本确定的。鉴别诊断试验确定了鳞状上皮细胞癌抗原水平能否将宫颈癌、肺癌或头颈癌与表观健康的、良性的或其他恶性群组区分开来。根据95%特异性下的鳞状上皮细胞癌抗原水平计算出鳞状上皮细胞癌抗原医学截断值。9个分析浓度的重复性变异系数 < 5.3%，中间精密变异系数 < 0.3%。方法学比较显示，与Architect和Kryptor系统具有很好的相关性（斜率分别为1.1和1.5）。表观健康人群的第95百分位数的参考区间为2.3 ng/mL（95%置信区间：1.9-3.8；欧洲队列，*n*=153）和2.7 ng/mL（95%置信区间：2.2-3.3；中国队列，*n*=146）。最佳的鉴别诊断结果见于宫颈鳞状上皮细胞癌：受试者工作特征曲线分析显示鳞状上皮细胞癌抗原水平（2.9 ng/mL的医学截断值）鉴别宫颈鳞状上皮细胞癌（*n*=127）与表观健康女性（*n*=286；曲线下面积：86.2%；95%置信区间：81.8-90.6；灵敏度：61.4%；特异性：95.6%），良性疾病（*n*=187；曲线下面积：86.3%；95%置信区间：81.2-91.3；灵敏度：61.4%；特异性：95.0%）和其他宫颈癌（*n*=157；曲线下面积：78.9%；95%置信区间：70.8-87.1；灵敏度：61.4%；特异性：86.7%）。鳞状上皮细胞癌还可帮助鉴别诊断肺癌。Elecsys鳞状上皮细胞癌检测技术在宫颈鳞状上皮细胞癌的临床实践中表现出了良好的性能，适合用于鉴别诊断。

## 概述

鳞状上皮细胞癌(SCC)是一种可源于不同类型组织的上皮性恶性肿瘤, 包括肺、宫颈和头颈部^[1]^。由于上皮细胞广泛分布在呼吸道和女性生殖道, 因此SCC在这些部位的发生率很高。非小细胞肺癌(NSCLC)约占所有肺癌的85%, 其中约35%的病例为SCC^[2, 3]^。此外, 约80%的宫颈癌和90%的头颈部癌病例是SCC^[4, 5]^。

鳞状上皮细胞癌抗原(SCCA)最早是从子宫中分离的, 且存在两种亚型: SCCA1和SCCA2^[6]^。两种亚型共表达于宫颈、舌头、扁桃体、食道和阴道的鳞状上皮中^[7]^。已发现SCCA的两种亚型在宫颈癌中升高^[8, 9]^。SCC患者血液中水平也会升高, 例如, 宫颈^[9, 10]^、肺^[11]^和头颈部SCC^[12, 13]^。而且还发现SCCA水平与所有这些SCC类型的肿瘤分期相关^[9, 10, 12-16]^。同样在肺、头颈部SCC中, SCCA1和SCCA2共表达于中度和分化良好的肿瘤^[7, 8]^。因此, 为了选择最佳临床灵敏度, 测定方法应该能够同时检测SCCA1和SCCA2, 以便确定总SCCA^[13]^。目前可用的测定系统均不能区分两种亚型, 原因是他们并非专为此目的而开发。

对于疑似疾病, 使用当前可用的免疫检测方法测量患者血清中的SCCA水平^[17-20]^。由于SCCA存在于唾液、毛发和皮屑而易于分布在气雾和尘埃中, 所以当前检测方法遭受污染的风险很高, 这可能导致SCCA检测值错误升高^[21]^。另外, 在良性肾脏、皮肤、肺和肝病患者中常见假阳性结果^[12, 22-24]^。SCCA水平对于血液采集的时机(麻醉前和麻醉后)和程序(静脉与动脉血管穿刺)也很敏感^[25]^。

以往研究的重点在于SCCA作用标志物用于宫颈癌^[26-31]^、肺癌^[32-34]^和头颈癌预后、监测和复发等用途^[35, 36]^。一些发表文献指出SCCA可用于组织学分型, 因为肺部SCC和宫颈SCC患者的SCCA浓度显著高于这些部位的腺癌患者^[37]^。然而, 关于SCCA用于鉴别SCC与对其他类型恶性肿瘤或良性疾病的证据有限, 而且其作用尚不明确^[13]^。为了帮助临床决策, 需要SCCA诊断医学截断值及其相关灵敏度和特异性指南。

该多中心研究首先评估了一种新型SCC测定方法的性能, 该方法开发用于测定SCCA1和SCCA2的血清水平。第二个目的是确定SCCA在宫颈、肺部和头颈部SCC中的分布模式, 并调查这种新型检测方法是否可用于鉴别诊断。

## 方法

### SCC测定

Elecsys^®^SCC测定法是一种电化学发光免疫测定法(ECLIA), 用于测量血清样本中的SCCA水平, 并用于Elecsys和cobas e分析仪。该测定方法使用以等摩尔方式识别人SCCA1和SCCA2两种亚型的SCC特异性单克隆抗体。它利用生物素-链霉素双抗夹心法原理, 然后通过电化学发光法读出数值。检测需要15 mL样本, 总检测周转时间为18 min。

### 研究中心和仪器

从2014年6月-2015年6月, 在阿姆斯特丹、巴塞罗那和波恩的三个欧洲研究中心评估了SCC测定方法的技术和临床性能。在四个欧洲研究中心和两个中国研究中心进行了临床评估(参考区间/鉴别诊断), 主要采用的是回顾性的病例对照研究设计。对于欧洲的参考区间, 测量的样本来自Kiel的样本库。中国研究中心(北京协和医院(PUMCH)和北京宣武医院)仅参加了2015年4月-6月的临床评估。所有使用Elecsys SCC测定方法的测量(包括性能和临床方面)均在cobas e 411或cobas e 601分析仪上进行。

### 研究符合伦理学标准

研究中所有涉及人类参与者的程序都符合机构和/或国家研究委员会的伦理标准, 并符合1964年赫尔辛基宣言及其后来的修正案或类似的伦理学标准。已经获取了研究中所有个体参与者的知情同意。

### 样本来源和处理

所有样本材料在测量后均保存在-20 ℃或-80 ℃下直至研究结束。样本在运输过程中均保持在-20 ℃或-80 ℃下。所有程序的实施均采用新的移液管吸头和容器, 从而尽量减少外部引起的样本污染。

### 性能评估

**精密度:**罗氏诊断公司为所有精密度实验提供样本材料, 这些试验根据临床和实验室标准协会(CLSI) EP 05-A3指南在欧洲三家研究中心进行^[38]^。重复性试验在阿姆斯特丹于21 d内完成。

**方法学比较:**该SCC测定方法与市售的Abbott Architect和Brahms Kryptor测定方法进行了比较。血清样本采集自常规临床实践, 并冷冻保存至测量。此外, 罗氏诊断公司还提供了日常临床实践中无法获得的高SCCA浓度的人血清样本和加标样本。对于每项比较, 使用*Passing-Bablok*回归来估计两个系统间的一致性。根据CLSI EP09-A3指南, 每个中心需要至少分析100个样本^[40]^。

### 临床评估

**参考区间确认:**来自单一研究中心(Kiel)的健康样本被用来确定欧洲队列的参考区间; 从Kiel研究中心的表观健康个体样本库中采集血清样本, 并且在波恩、阿姆斯特丹和巴塞罗那测定SCCA水平。

对于中国人群参考区间, 表观健康个体样本采集自北京的PUMCH和宣武医院。所有样本均在PUMCH研究中心测定。

患者招募基于健康检查和调查问卷, 参考区间人群的最终入组基于临床生化参数, 包括血糖、胆碱酯酶、肌酐、C-反应蛋白(CRP)和血红蛋白水平, 均在Kiel测定。如果这些临床化学结果异常, 则排除这些个体。确定90%置信区间(CI)的参考区间需要至少120个样本, 95%CI分析需要146个样本。

**鉴别诊断:**该研究的临床部分的重点是确定能否单纯利用SCCA水平来鉴别宫颈、肺部或头颈部癌症与表观健康、良性或其他恶性肿瘤队列。在巴塞罗那、波恩、阿姆斯特丹和北京, 从本地血清库采集假名化的样本, 并用于鉴别诊断实验。在各自的欧洲采集点测定欧洲样本, 并在PUMCH测定所有中国样本。在源数据验证过程中检查确认是否提供了知情同意或弃权证书。患者样本并非在手术或麻醉前采集, 这是样本库的常规程序。计算鉴别诊断实验的患者样本大小, 作为允许进行基础描述性分析所需的最小样本量。

鉴别诊断所需表观健康人群的主要入选标准包括临床上表观健康及正常的常规临床生化结果(包括胆红素、CRP、丙氨酸转氨酶、天冬氨酸转氨酶以排除炎症、肾脏或肝脏疾病); 没有已知的肿瘤疾病史或严重的肺部良性疾病史; 无急性肺部疾患; 未接受与所排除疾病/功能紊乱相关的药物; 记录到吸烟习惯。样本并非来自献血者, 且必须从不同种族获取。

对于恶性肿瘤队列, 要求患者患有原发性肿瘤, 但尚未接受治疗或手术。必须提供肿瘤淋巴结转移(TNM)和国际抗癌联盟(UICC)分期信息^[41]^。

一般排除标准如下:年龄＜18岁, 当前怀孕和其他恶性疾病病史。由于晚期慢性肾病(CKD)患者的SCCA水平较高, 因此除了分析良性肾病外, 所有分析均排除肾小球滤过率(GFR)＜30 mL/min/1.73 m^2^(CKD 4和5期)的样本^[23, 24, 42, 43]^。而选择GFR阈值为30 mL/min/1.73 m^2^是因为发现GFR较低的患者存在CKD 4期和5期(即晚期)疾病^[44]^。在表观健康、良性和恶性肿瘤队列中评估SCCA水平对于年龄、性别、部位、种族、吸烟状况和UICC分期的作用。

### 统计分析

使用基于Windows的计算机辅助评估(WinCAEv)软件记录下测定数据^[45]^。使用Medrio和MACRO软件收集临床数据。使用版本3.0.1的R软件和版本9.3的统计分析软件(SAS)进行了所有使用临床和技术信息的统计分析。

排除/替换异常值后, 利用所有数据分析了技术实验。所有研究中心都采用了一致的异常值识别和处理方法。对于精密度实验, 使用SAS(版本9.3)和R (版本3.0.1, 附加的软件软件包方差组分分析(VCA)版本1.0.6)计算重复性、中间精密度和再现性, 并进行了异常值检测, 上述程序均依照CLSIEP05-A3指南描述^[38]^。使用*Passing-Bablok*回归分析和*Pearson*相关系数来评估符合CLSI EP09-A3.36的方法学比较研究的相关性^[36]^。对于方法学比较, 未采用异常值检测方法。

根据国际临床化学和检验医学联合会的参考区间确定建议, 对参考区间进行了测定和统计分析^[46]^。

患者特征的描述性统计。为了计算不同群组的SCCA水平的参考区间和SCCA测定值, 我们使用了基于Hahn和Meeker^[47]^方法的非参百分位数CI。计算了用于鉴别诊断分析的受试者工作特征曲线(ROC)和曲线下面积(AUC)。基于这些结果, 计算95%特异性下的灵敏度; 这里95%的特异性根据以往关于宫颈癌的文献中检查的方法来选择^[48]^。其医学截断值基于用于对比的95%特异性下的SCCA水平。

从ROC分析中排除了源自巴塞罗那研究中心的表观健康和良性头颈部队列的患者数据, 因为这些患者意外表现为SCCA阳性。对于这类SCCA水平升高, 没有发现明确的根本原因, 但在研究中心进行的内部实验确认这些水平可能是由于已知的污染风险, 例如唾液和尘埃, 正如以往发表文献中的报道^[21]^。

## 结果

### 技术评估

**精密度:** 表 1列出了所有研究中心的再现性、中间精密度和重复性结果。对于平均值＞1.8 ng/mL的样本, 应使用变异系数(CV)来评估精密度:对于＞1.8 ng/ mL-5 ng/mL的样本CV≤5%, 对于＞5 mg/mL-70 mg/mL的样本CV≤6.5% mL, 规定为可接受的中间精密度; 而对于＞1.8 mg/mL-5 ng/mL的样本, CV≤10%规定为可以接受的再现性。在阿姆斯特丹研究中心, 21 d内进行的再现性研究的CV值在4.3%-8.0%范围内; 其中间精密度范围是在4.3%-7.0%之间; 重复性CV≤3.8%。对于平均值≤1.8 ng/mL的样本, 应使用标准差(SD)来评估精密度, 并将SD ≤0.2规定为可以接受。再现性和中间精度的范围是SD 0.1-0.2, 重复性是SD 0.0-0.1。

**1:** 全部三个研究中心的SCC测定的精密度结果^a^

样本SCC1	平均值（ng/mL)	重复性			中间精密度			再现性	
		SD (ng/mL)	CV (%)		SD (ng/mL)	CV (%)		SD (ng/mL)	CV (%)
SCC1	2.2	0.1	3.8		0.2	7.0		0.2	8.0
SCC2	22.7	0.6	2.6		1.3	5.6		1.4	6.1
HSP 01	0.6	0.0	5.3		0.1	10.3		0.1	14.0
HSP 02	1.5	0.1	4.4		0.1	7.7		0.1	8.7
HSP 03	0.2	0.1	4.1		0.2	6.3		0.2	7.0
HSP 04	35.6	1.2	3.4		2.0	5.5		2.3	6.4
HSP 05	60.4	1.8	3.0		4.0	6.6		4.3	7.1
HSP 06	3.5	0.1	3.7		0.2	5.7		0.2	6.8
HSP 07	35.0	0.7	2.0		1.5	4.3		1.5	4.3
SCC:鳞状上皮细胞癌；SD:标准差；CV:变异系数；HSP:人血清池。^a^从符合CLSI EP05-A3的分析中排除了三个异常值。									

**方法学比较:**经计算, cobas e 411相对于Architect的斜率为1.13(95%CI: 1.04-1.20), 截距为0.15(95%CI: 0.11-0.37)(图 1(a)), *Pearson*相关系数*r*=0.937。对于cobas e 601和Kryptor系统间的对比, 斜率为1.50(95%CI: 1.44-1.54), 截距为0.48(95%CI: 0.40-0.56), *Pearson*相关系数*r*=0.988(图 1(b))。

**1:**
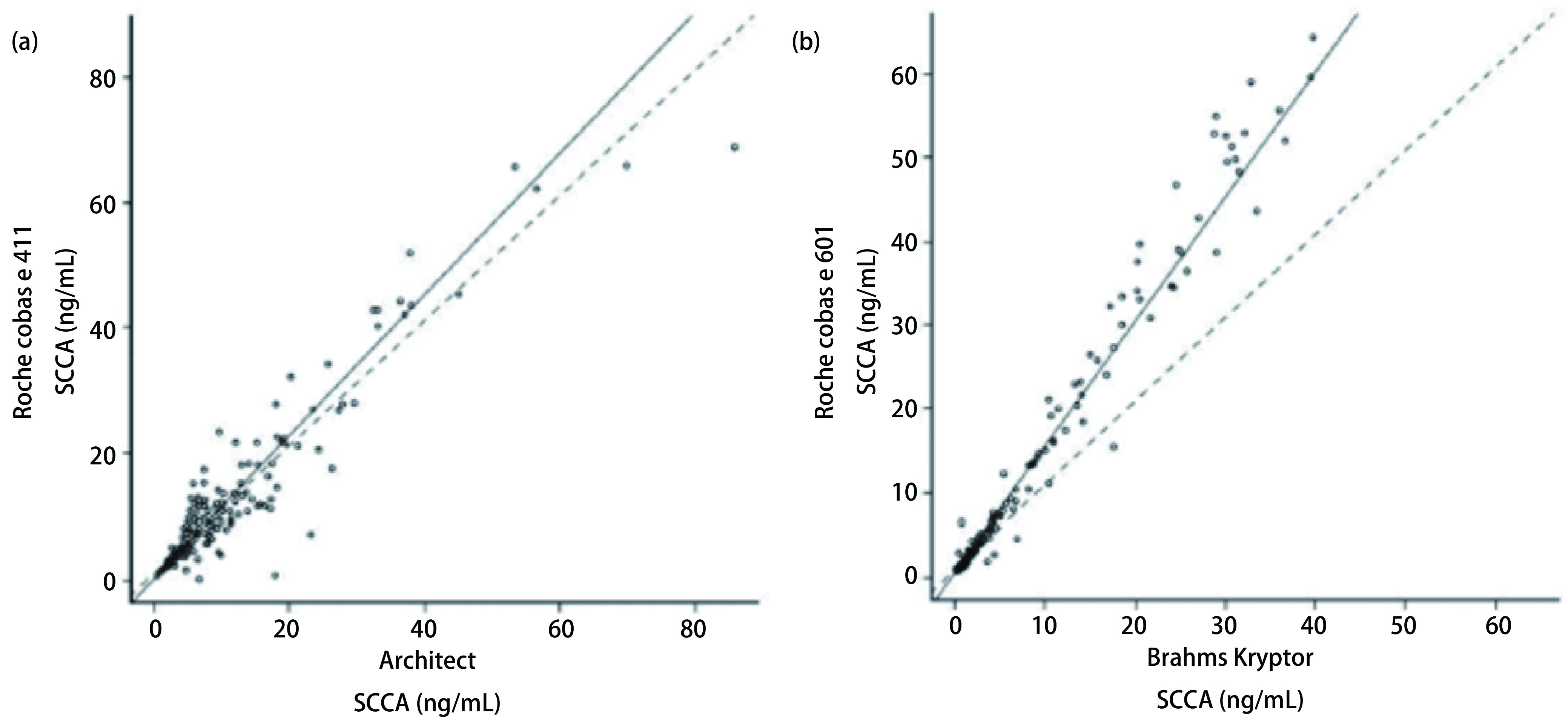
鳞状上皮细胞癌抗原（SCCA）测定方法学比较结果。(a)在巴塞罗那研究中心采用血清样本对比cobas e 411和Architect系统(*n*=193)；(b)在阿姆斯特丹研究中心采用血清样本对比cobas e 601和Kryptor系统（*n*=180）。

### 临床评价

**参考区间确认:**参考区间分析一共包括了153例表观健康的欧洲个体(表 2)。表观健康个体中SCCA的中位数浓度为1.1 ng/mL。第95百分位数和第97.5百分位数分别为2.3 ng/mL(95%CI: 1.9-3.8)和2.7 ng/mL(95%CI: 2.2-4.4)。

**2:** 参考和鉴别诊断人群的患者人口统计学

	参考区间人群			鉴别诊断人群						
	欧洲 (*n*=153)	中国人 (*n*=146)		全体 (*N*=2, 039)	表观健康 (*n*=246)	良性 (*n*=444)	肺癌 (*n*=802)	头颈癌 (*n*=160)	宫颈癌 (*n*=157)	其他恶性肿瘤 (*n*=230)
年龄，平均岁（SD)	49.5 (17.3)	38.5(13.6)		56.7(15.4)	41.5 (14.5)	53.2 (17.5)	62.3(11.4)	60.8 (10.7)	51.6 (14.5)	61.1 (13.7)
男性，*n* (%)	75 (49)	55 (38)		1034 (50.7)	87 (35.4)	208 (46.9)	498(62.1)	121 (75.6)	-	120 (52.2)
种族，*n* (%)										
亚洲-中国人	-	146(100)		479 (23.5)	146 (59.4)	91 (20.5)	207 (25.8)	0 (0.0)	4(2.6)	31 (13.5)
黑人	-	-		1 (0.1)	0 (0.0)	0 (0.0)	0 (0.0)	1 (0.6)	0 (0.0)	0 (0.0)
高加索/白种人	153(100)	-		844(41.4)	50 (20.3)	353 (79.5)	129(16.1)	135 (84.4)	110 (70.1)	67 (29.1)
未指定	-	-		715(35.1)	50 (20.3)	0 (0.0)	466 (58.1)	24(15.0)	43 (27.4)	132 (57.4)
吸烟习惯，*n* (%)										
现吸烟者	29(19)	14 (9.6)		708 (34.7)	17 (6.9)	60 (13.5)	449 (56.0)	86 (53.4)	37(23.6)	59(25.6)
过去吸烟者	17(11.1)	6 (4.1)		318 (15.6)	6(2.4)	38 (8.6)	211 (26.3)	19(1 1.9)	8 (5.1)	36 (15.7)
从未吸烟者	107 (69.9)	126 (86.3)		478 (23.4)	135 (54.9)	100(22.5)	107(13.3)	14 (8.8)	50 (31.9)	72(31.3)
未指定	-	-		535 (26.2)	88 (35.8)	246 (55.4)	35 (4.4)	41 (25.6)	62 (39.5)	63 (27.4)
分期, *n* (%) ^b^							215	154(100)	127(100)	-
0	-	-		-	-	-	-	10 (6.5)	-	-
1	-	-		-	-	-	21 (9.8)	14 (9.1)	31 (24.4)	-
Ⅱ	-	-		-	-	-	19 (8.8)	13 (8.4)	45 (35.4)	-
Ⅲ	-	-		-	-	-	131 (60.9)	24(15.6)	34 (26.8)	-
Ⅳ	-	-		-	-	-	39 (18.1)	92 (59.7)	16 (12.6)	-
N/A	-	-		-	-	-	5 (23.2)	1 (0.6)	1 (0.8)	-
SD:标准差；CKD:慢性肾脏疾病；SCC:鳞状上皮细胞癌。^a^排除了CKD4和5(除了良性肾病患者）的患者和巴塞罗那表观健康队列。^b^Staging信息仅适用于SCC患者。										

对于中国参考区间队列, 分析了146位个体的样本。中位SCCA水平为1.1 ng/mL, 第95百分位数为2.7 ng/mL(95%CI: 2.2-3.3), 第97.5百分位数为3.0 ng/mL (95%CI: 2.5-4.1)(表 3)。

**3:** 不同队列的SCCA水平

参考区间人群	*N*	SCCA中位数(ng/mL)	95%百分位数(95%CI), 97.5%百分位数(95%CI)(ng/mL)
欧洲队列	153	1.1	2.3 (1.9-3.8), 27(2.2-4.4)
中国队列	146	1.1	2.7 (2.2-3.3), 3.0 (2.5-4.1)
鉴别诊断人群	*N*	SCCA中位数(ng/mL)	IQR (ng/mL)
表观健康	246	1.2	0.8-1.6
表观健康女性	159	1.1	0.8-1.6
表观健康男性	87	1.3	1.0-1.8
宫颈	157	3.5	1.4-14.7
SCC	127	7.4	1.6-19.7
腺癌	18	1.2	0.9-1.6
其他	12	1.7	1.4-2.6
肺^a^	802	1.2	0.8-2.1
NSCLC全部	608	1.4	0.9-2.4
NSCLC SCC	215	1.9	1.2-4.3
NSCLC腺癌	261	1.0	0.7-1.7
NSCLC排除混合性/未特指	18	1.4	0.9-3.1
SCLC全部	189	1.0	0.7-1.5
其他	5	^b^	^b^
头颈部	160	1.9	1.3-2.9
SCC	154	2.0	1.3-2.9
腺癌	2	2.3^b^	2.3-3.6^b^
其他	4	0.9^b^	0.7-1.0^b^
良性疾病	444	1.4	1.0-2.3
宫颈	60	1.2	0.9-1.4
肺	123	1.1	0.8-1.6
头颈部	69	1.9	1.4-3.5
其他	192	1.7	1.1-2.7
肾脏	44	2.3	1.5-4.2
恶性肿瘤其他	290	1.2	0.8-1.9
IQR:四分位间距；CI:置信区间；NSCLC:非小细胞肺癌；SCCA:鳞状上皮细胞癌抗原；SCC:鳞状上皮细胞癌；SCLC:小细胞肺癌；CKD:慢性肾脏疾病。^a^排除CKD 4和5(不包括肾脏）。^b^样本太小而无法进行有意义的比较。			

**鉴别诊断:患者人群**。表 2给出鉴别诊断人群的患者人口统计学资料。其年龄中位数为56.7岁(范围: 15.4), 男女比例几乎相等。至少有一半的患者是当前(34.7%)或既往(15.6%)吸烟者。我们调查了不同队列中研究中心、年龄、性别、种族和吸烟对于SCCA水平的影响, 但未发现重要影响(数据未显示)。在所有队列中, 男性的SCCA水平趋向于略高于女性, 但仅为临界性差异(重叠的四分位间距(IQR):健康男性:=1.0 ng/mL-1.7 ng/mL; 健康女性: 0.8 ng/mL-1.5 ng/mL), 极可能没有临床相关性, 虽然有可能是队列组成的结果。

**鉴别诊断:宫颈癌**。相较于表观健康的女性[1.06 (IQR: 0.8-1.6) ng/mL]、良性妇科疾病患者[1.2(IQR: 0.9-1.4) ng/mL]和其他宫颈癌患者[1.7(IQR: 1.4-1.9) ng/mL], 宫颈SCC患者有着显著较高的SCCA中位数水平[7.4(IQR: 1.6-19.2) ng/mL](表 3)。

ROC分析显示宫颈SCC与表观健康的女性鉴别, 其AUC为86.2%(95%CI: 81.8-90.6);与良性妇科疾病鉴别, AUC为86.3%(95%CI: 81.2-91.3);与其他宫颈癌鉴别, AUC为78.9%(95%CI: 70.8-87.1)(图 2(a))。

**2:**
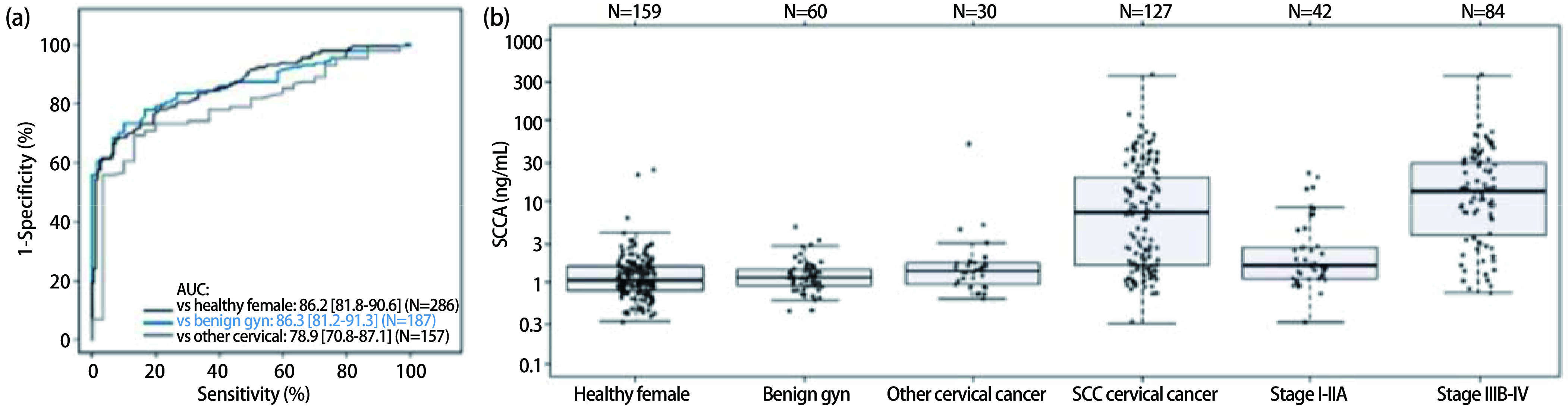
SCCA用于宫颈癌的鉴别诊断。(a)SCCA水平作为鉴别宫颈SCC与其他患者队列的诊断生物标志物的ROC分析。(b)健康女性和良性妇科疾病患者、其他宫颈癌、SCC宫颈癌、Ⅰ期-ⅡA期和ⅡV期-Ⅳ期患者的SCCA水平。其他宫颈癌组包括腺癌患者（*n*=12）和其他宫颈癌患者（*n*=18）。女性仅来自表观健康的个体组（*n*=159）。对于按照Ⅰ期-ⅡA期和ⅡB期-Ⅳ期的细分，*n*=1例遗漏患者由于没有分期信息而未显示。

对于宫颈SCC和健康妇女间的鉴别, 特异性在95%时的灵敏度为61.4%, 相较于良性患者的灵敏度为61.4%, 而相较于其他宫颈癌的灵敏度为55.9%(表 4)。

**4:** ROC分析AUC、灵敏度和特异性结果

	AUC, % (95%CI)	灵敏度, % (95%CI)	特异性, % (95%CI)	95%时特异性灵敏度, % (95%CI)
宫颈		截断值为2.9 ng/mL		
SCC *vs*表观健康	86.2 (81.8-90.6)	61.4 (52.4-69.9)	95.6 (91.1-98.2)	61.4 (52.4-69.9)
SCC *vs*良性宫颈疾病^a^	86.3 (81.2-91.3)	61.4 (52.4-69.9)	95.0 (86.1-99.0)	61.4 (52.4-69.9)
SCC *vs*其他子宫颈癌	78.9 (70.8-87.1)	61.4 (52.4-69.9)	86.7 (69.3-96.2)	55.9 (46.8-64.7)
肺		截断值为3.4 ng/mL		
SCC *vs*表观健康	72.5 (67.8-77.2)	28.4 (22.5-34.9)	98.4 (95.9-99.6)	34.4 (28.1-41.2)
SCC *vs*良性肺病^a^	73.8 (68.6-79.1)	28.4 (22.5-34.9)	96.7 (91.9-99.1)	39.1 (32.5-45.9)
SCC *vs*其他NSCLC^b^	71.7 (67.2-76.2)	28.4 (22.5-34.9)	91.4 (87.5-94.4)	17.7 (12.8-23.4)
头颈部		截断值为2.8 ng/mL		
SCC *vs*表观健康	74.2 (69.2-79.1)	26.6 (19.8-34.3)	95.5 (92.1-97.7)	26.6 (19.8-34.3)
SCC *vs*良性头颈部疾病^a^	67.5 (59.5-75.6)	26.6 (19.8-34.3)	100.0 (91.0-100.0)	27.9 (21.0-35.7)
SCC *vs*其他头颈部癌症	^c^	^c^	^c^	^c^
AUC:曲线下面积; Cl:置信区间; ROC:受试者工作特征曲线; NSCLC：非小细胞肺癌; SCC:鳞状上皮细胞癌; NOS:未特指。 ^a^良性疾病包括肺、头颈部、妇科疾病。 ^b^排除NOS/混合型。 ^c^样本量太小，无法进行有意义的分析。				

对于所有宫颈鉴别诊断, 基于95%特异性的SCCA医学截断值为2.9 ng/mL。在此医学截断值水平下, 相比于表观健康的女性, 检测宫颈鳞癌的灵敏度为61.4%, 特异性为95.6%(表 4)。因此, 95.6%的表观健康女性的SCCA水平≤2.9 ng/mL, 而61.4%的宫颈SCC患者的SCCA水平＞2.9 ng/mL。由于与良性宫颈癌和其他宫颈癌的比较, 每种适用用途的95%特异性的医学截断值水平非常相似, 每种适应用途均应用2.9 ng/mL的医学截断值。因此, 使用相同的SCCA医学截断值(2.9 ng/mL), 对比良性疾病检测宫颈SCC的灵敏度为61.4%, 特异性为95.0%。2.9 ng/mL的SCCA水平可用于鉴别宫颈SCC和其他宫颈癌, 其灵敏度为61.4%, 特异性为86.7%。

值得注意的是, 与早期宫颈癌(Ⅰ期-ⅡA期; 图 2(b))相比, 较晚UICC分期的宫颈癌(ⅡB期-Ⅳ期)患者有着显著更高的中位SCCA水平。

**鉴别诊断:肺癌**。相较于健康个体[1.2(IQR: 0.8-1.6) ng/mL], 其他NSCLC患者[1.0(IQR: 0.7-1.7) ng/mL]和良性肺病患者[1.1(IQR: 0.8-1.6) ng/mL], NSCLC SCC队列患者存在轻微且非显著性较高的SCCA中位水平[1.9(IQR: 0.9-2.4) ng/mL](图 3(a)和表 3)。

**3:**
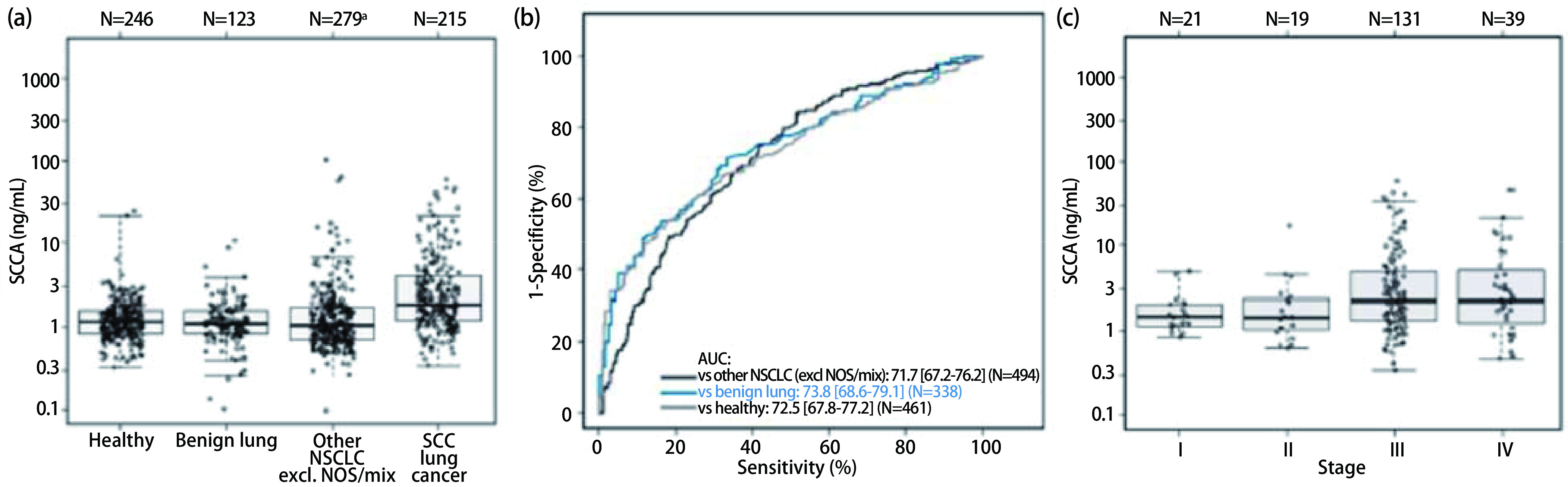
SCCA用于肺癌的鉴别诊断。(a)肺SCC对比表观健康患者、良性疾病和其他类型肺癌患者的SCCA水平。(b)SCCA水平作为鉴别肺SCC与其他患者队列的诊断生物标志物的ROC分析。(c)肺SCC UICC分期的SCCA水平。

ROC分析显示SCCA可用于肺癌与表观健康个体, 其AUC为72.5%(95%CI: 67.8-77.2);良性肺部疾病, 其AUC为73.8%(95%CI: 68.6-91.3); NSCLC SCC患者和其他NSCLC类型患者间的鉴别[不包括混合型/未特指(NOS)], AUC为71.7%(95%CI: 67.2-76.2;图 3(b))。

在特异性为95%时, 肺SCC和健康个体比较的灵敏度为34.4%, 与良性患者比较时的灵敏度为39.1%, 而与其他NSCLC鉴别时的灵敏度为17.7%(表 4)。

对于所有肺部鉴别诊断, 基于95%特异性的相关SCCA医学截断值为3.4 ng/mL。使用3.4 ng/mL的SCCA医学截断值, 可鉴别NSCLC SCC和表观健康的患者队列, 灵敏度和特异性分别为28.4%和98.4%。因此, 98.4%的表观健康个体的SCCA水平≤3.4 ng/mL, 而28.6%的NSCLC SCC患者的SCCA水平＞3.4 ng/mL。此外, 针对与良性肺癌和其他NSCLC的鉴别, 3.4 ng/mL的医学截断值显示了＞95%的特异性, 因此认为适合采用一致的医学截断值水平。相同的SCCA医学截断值能够鉴别NSCLC SCC和良性肺疾病队列, 其灵敏度和特异性分别为28.4%和96.7%, 以及鉴别NSCLC SCC和其他NSCLC患者, 灵敏度为28.4%, 特异性为91.4%。

较晚期的NSCLC SCC患者具有更高的SCCA水平, 尤其是Ⅲ-Ⅳ期[2.2(IQR: 1.3-5.1) ng/mL; *P*＜0.001](图 3(c))。

**鉴别诊断:头颈癌**。相较于表观健康个体[1.2 (IQR: 0.8-1.6) ng/mL](差别无显著性), 头颈部SCC队列患者存在稍高的SCCA中位水平[2.0(IQR: 1.3-2.9) ng/mL], 且该水平与良性头颈部疾病患者相似[1.9 (IQR: 1.4-3.5) ng/mL; 表 3]。然而, 头颈部SCC的中位SCCA水平与各对照组间的差异不如其他两种恶性肿瘤(即NSCLC SCC和颈部SCC)那样明显。头颈部SCC队列中SCCA水平(2.0 ng/mL)高于其他组织学类型的头颈部癌症患者(0.9 ng/mL)。

ROC分析显示, 用于鉴别头颈部SCC与表观健康个体的AUC为74.2%(95%CI: 69.2-79.1)。头颈部SCC可与良性疾病鉴别, 其AUC为67.5%(95%CI: 59.5-75.6)。但非SCC的头颈部癌症队列的患者人数过少, 无法进行有意义的ROC分析, 因此难以解释结果。

在特异性为95%时, 头颈部SCC和健康个体间比较的灵敏度为26.6%, 而与良性头颈部患者间比较时的灵敏度为27.9%(表 4)。

采用＞2.8 ng/mL的SCCA鉴别医学截断值, 头颈部SCC与表观健康患者间比较的灵敏度为26.6%, 特异性为95.5%。使用相同的医学截断值, 相对于良性头颈部疾病检出头颈部SCC的灵敏度为26.6%(95%CI: 19.8-34.3), 特异性为100.0%。

头颈部恶性肿瘤组中SCCA水平和UICC分期间没有明确的相关性(数据未显示)。

## 讨论

这项多中心研究评估了以等摩尔方式测定SCCA1和SCCA2血清水平的新型SCC测定方法的技术性能, 这是第一种可同时测定SCCA1和SCCA2的测定方法。目前, 关于SCCA生物标志物作为SCC患者鉴别诊断工具实用性的研究很有限。因此, 还评估了这种生物标志物用于颈部、肺部和头颈部SCC鉴别诊断的临床用途。目前最大规模的一项研究, 其中包括了逾2, 000份来自不同队列的患者。

总体而言, Elecsys SCC检测方法表现出了良好的精密度, 且与其他商业化检测方法一致^[17]^。从精密度结果中删除了20个异常值; 这些可能是由于皮肤或唾液污染问题造成, 正如SCCA中常见的问题^[21]^。按照CLSI EP05-A3异常值分析方法去除了3个异常值^[38]^。即使包括这些异常值, Elecsys SCC测定方法的技术性能结果也与最常采用的方法, 即Architect检测方法有良好的相关性。尽管抗体在检测SCCA1和SCCA2两种亚型的能力方面存在差异, 但其技术性能类似。应该指出的是, 当实验室改变方法时, 必须要考虑到与Kryptor方法相比时出现的1.5的斜率。Elecsys SCC ECLIA是唯一一种以等摩尔方式检测SCCA1和SCCA2的方法; 这可能是出现这种差别的原因。另外, 使用不同方法时可能会出现个体差异。因此, 只有在使用相同的方法进行连续测定时才能解释标志物动力学。

同时对来自健康患者的欧洲和中国样本进行了分析, 以便确定高加索人群和中国人群的准确参考区间。欧洲队列的中位值为1.1 ng/mL(第95百分位数: 2.3 ng/mL), 中国人队列为1.1 ng/mL(第95百分位数: 2.7 ng/mL)。这些都与以前研究的估计值相似: Architect在美国患者群体中见到了2.1 ng/mL的第95百分位数^[20]^或略高于其他报告值^[13]^。

正如先前所见, 宫颈SCC患者群体的SCCA水平远高于其他人群^[9, 10, 13]^。肺或头颈部SCC队列中的SCCA水平并未高于先前观察到的结果^[11, 13]^。性别、年龄、种族和吸烟状况对SCCA水平没有显著影响。这一点在表观健康队列中也是如此。

对于将SCCA用作鉴别诊断工具, 在宫颈SCC队列中观察到了最强结果。当灵敏度为95%时, 鉴别健康队列和良性疾病队列的特异性为61.4%, 而宫颈恶性肿瘤的特异性为55.9%。我们认为, 采用一个医学截断值来鉴别宫颈SCC患者和其他患者是很有效的:为了取得SCCA诊断宫颈SCC时95.6%的特异性, 采用了2.9 ng/mL作为医学截断值。因此, 大约96%的患者中, 单纯SCCA水平或能够正确鉴别宫颈SCC患者与表观健康个体或良性疾病患者。对于86.6%的患者, ≤2.9 ng/mL的SCCA能够区分宫颈癌和其他类型的宫颈癌。2010年发布的最新国家临床生物化学协会指南指出, 所有病例的宫颈SCC诊断只应通过组织病理学检查结果来确定; 然而, 我们的研究结果表明, SCCA可作为灵敏的宫颈SCC鉴别诊断工具^[13]^。

较高的SCCA水平见于较晚期的宫颈SCC, 这与先前的研究结果一致^[13]^。从Ⅰ期-ⅡA期至ⅡB期-Ⅳ期, 宫颈癌的SCCA水平大幅增加具有临床相关性, 应进一步开发用于早期癌症检测。ⅡA期和ⅡB分期间的这种大幅度增加还见于既往研究中得报道^[9, 10, 13, 16, 30, 49]^。而ⅡA期和ⅡB期间的这种增加令人关注, 基于这些结果, 单纯检测血液SCCA水平有可能成为两个分期间的鉴别因子。从历史上看, ⅡA期仍被认为是“早期”, 而前期手术便是一种治疗选择^[50]^。然而, ⅡB期及更高分期均属于临床晚期, 主要治疗是放化疗。此外, 宫颈癌在资源有限(例如探查性腹腔镜检查或高级成像)的低收入国家非常普遍, 因此SCCA水平可能是一种有效且便宜的快速附加诊断检测方法, 可用于患者分层和有限资源的更好分配。沿着患者临床路径, 可以设想SCCA使用的连续性。对于临床妇科检测结果未定的患者, SCCA水平升高会增加对于宫颈癌的怀疑, 并防止进一步延误治疗。由于大约10%的宫颈癌并非SCC类型, 因此SCCA水平可用于鉴定这些未分类的宫颈恶性肿瘤患者。此外, 对于诊断为宫颈SCC的患者, 较高的SCCA水平会怀疑晚期疾病, 因此可考虑进行额外的分期测试, 例如通过骨扫描排除骨转移。

对于肺癌患者, SCCA有着中等的鉴别SCC和表观健康、良性和SCLC类型的能力。值得注意的是, 异常SCCA水平提示有较高的NSCLC可能性, 特别是SCC^[51, 52]^。因此, 对于肺癌患者, SCC可能与其他生物标志物联用时最有效。例如癌胚抗原(CEA)、细胞角蛋白19片段(CYFRA 21-1)、神经元特异性烯醇化酶(NSE)或前胃泌素释放肽前体(ProGRP) ^[51, 53]^。目前正在进一步研究其与其他多种标志物的联合使用。

先前研究显示头颈部癌患者的SCCA水平升高, ^[36, 54]^且较晚期疾病患者的SCCA水平更高^[12, 35]^。然而, 我们发现, 相较于良性疾病患者, 头颈部SCC患者的SCCA水平差别很小。另外, 在TNM分期中没有观察到明确的分布模式。SCCA鉴别头颈部癌症与表观健康患者或良性疾病患者的能力很低, 在95%特异性下, 灵敏度分别为26.6%和27.9%。这些结果表明, SCCA作为单一标志物无法有效地用于头颈部癌症的分期或鉴别诊断。

该研究的潜在局限性包括使用血库中的样本, 并采取一些预防措施来尽量减少可能的污染风险, 这可能并不能完全代表临床实验室的日常工作。

总之, 这项多中心研究表明Elecsys SCC测定方法在临床实践中有着良好的SCCA测定技术性能, 并且与其他两种商业化的测定方法的性能相当。据我们所知, 这是SCCA用于宫颈、肺部和头颈部SCC鉴别诊断的最大规模研究之一, 其中包括了来自两大洲的对照组。结果显示SCCA水平可有效用于SCC类型的鉴别诊断, 特别是用于宫颈癌患者, 而且可能是宫颈SCC患者的简单诊断工具。

## 致谢

作者要感谢德国Kiel, Laboratorium fur Klinische Forschung GmbH的Wolfgang Junge, 为采集参考区间样本作出的贡献, 并感谢荷兰阿姆斯特丹荷兰癌症研究所的Jeroen de Jong提供的临床意见。德国, Penzberg罗氏诊断有限公司的Birgit Wehnl提供了研究管理支持和建议。Monika Steclik(Triga-S.e.K., Habach, 德国)和Silvia Barbara Schmid(罗氏诊断公司, Penzberg, 德国)对研究进行了监督。德国Penzberg罗氏诊断公司的Christina Rabe和Katharina Buck提供了统计支持和建议。Marcus-Rene Lisy(德国Penzberg罗氏诊断公司)提供了科学咨询。Kim Brown(瑞士, Rotkreuz, 罗氏诊断国际有限公司)为该稿件提供了医学写作帮助。Stefan Holdenrieder、Rafael Molina、Ling Qiu、Xiuyi Zhi、Sandra Rutz和Catharina M Korse参与了数据收集、数据解释和稿件撰写, 并批准了稿件提交。Christine Engel、Pia Kasper-Sauer和Farshid Dayyani参与了数据解释、稿件撰写并批准稿件提交。

## 研究符合伦理学标准

研究中所有涉及人类参与者的程序都符合机构和/或国家研究委员会的伦理标准, 并符合1964年赫尔辛基宣言及其后来的修正案或类似的伦理学标准。已经获取了研究中所有个体参与者的知情同意。

## 利益冲突声明

作者声明以下与研究、作者身份和/或文章出版有关的潜在利益冲突: Sandra Rutz、Christine Engel和Pia Kasper-Sauer是罗氏诊断公司的雇员。在研究时, Farshid Dayyani是罗氏诊断公司的雇员。Stefan Holdenrieder、Rafael Molina和Catharina M Korse获取了罗氏诊断公司提供的资助/研究支持。

## 资助

作者声明收到以下与研究、作者身份和/或文章出版有关的财务支持:该研究由罗氏诊断公司资助。Gardiner-Caldwell Communications的Emma McConnell提供了医学写作支持。本研究由罗氏诊断公司主办。
